# Long-standing insulinoma: two case reports and review of the literature

**DOI:** 10.1186/s13104-015-1424-1

**Published:** 2015-09-15

**Authors:** Mohamed Tarchouli, Abdelmounaim Ait Ali, Moulay Brahim Ratbi, Mohamed said Belhamidi, Mohamed Essarghini, El Mehdi Aboulfeth, Mohamed Bouzroud, Yassir Sbitti, Mohamed Oukabli, Mohammed Elfahssi, Khalid Sair

**Affiliations:** Department of Digestive Surgery, Faculty of Medicine and Pharmacy, Mohammed V Military Hospital, Mohammed V University, Rabat, Morocco; Department of Oncology, Faculty of Medicine and Pharmacy, Mohammed V Military Hospital, Mohammed V University, Rabat, Morocco; Department of Pathology, Faculty of Medicine and Pharmacy, Mohammed V Military Hospital, Mohammed V University, Rabat, Morocco

**Keywords:** Insulinoma, Pancreas, Diagnosis, Management, Surgery

## Abstract

**Background:**

Insulinomas are rare pancreatic endocrine tumors. Most are benign and solitary. However, the nonspecific symptoms and small size of these tumors led to difficulties of diagnosis and localization.

**Case presentation:**

We present two Arab patients with pancreatic long-standing insulinoma. Both patients presented episodic hypoglycemic symptoms respectively during 10 and 2 years. Biochemical and morphological workup detected localized pancreatic insulinoma. Open procedure surgery was done for the two patients and insulinomas were successfully removed by enucleation.

**Conclusion:**

Insulinoma remains a diagnostic challenge to practitioners. Diagnosis of suspected cases is easily confirmed by standard endocrine tests, especially the supervised fasting test. Accurate preoperative localization is essential for more effective and safest surgery.

## Background

Among pancreatic endocrine tumors, insulinoma is the most common type. This tumor was reported in 1–4 people per one million person years [1]. It can be seen at any age and occurs slightly more frequently in female than male [2]. Usually insulinoma clinical presentation is benign, with a solitary and small size (<2 cm in diameter). Most are sporadic, however, 10 % of insulinomas are multiple and occur as part of multiple endocrine neoplasia syndrome type I (MEN–I). Because of nonspecific symptoms, insulinoma may be misdiagnosed with other disorders. Patients often present with hypoglycemia signs resulting from inappropriate insulin secretion [3]. After biochemical confirmation of hyperinsulinism, preoperative localization of the tumor in the pancreas may be difficult. Surgical removal, often curative, continues to be the treatment of choice.

In this manuscript, we report 2 cases of pancreatic insulinoma, and we discuss diagnosis, localization and management of this uncommon disease.

## Case presentation 1

**Fig. 1:**
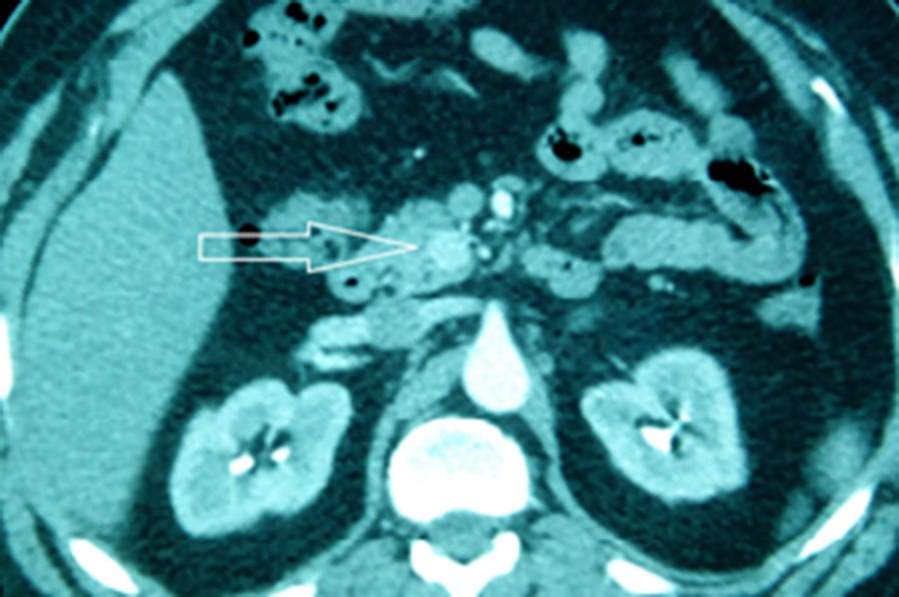
Axial computed tomography scan image: mass in the uncinate process of pancreas measuring 15 mm in diameter with bright contrast enhancement during arterial phase (*arrow*), without pathologic intra- abdominal lymph nodes

A 60-year-old Arab male had history of episodic and repetitive symptoms including diaphoresis, tremors, palpitations, and occasional loss of consciousness since 10 years. These symptoms, associated with a weight gain and a chronic weakness, occurred especially at night away from meals and were relieved with eating something. He had no family history of endocrine disease. Physical examination showed a healthy man with a Body Mass Index (BMI) of 34.9 kg/m^2^. Blood laboratory tests demonstrated a low initial glucose level at 46 mg/dl (70–11 mg/dl), a high plasma insulin level at 70.4 μIU/ml (2.6–24.9 μIU/ml), and a high C-peptide level at 6.76 ng/ml (0.8–4.2 ng/ml). Prolonged supervised fasting test was applied and produced symptomatic hypoglycemia with hyperinsulinemia. Urine for sulfonylurea screen was negative. Abdominal computed tomography (CT) scan with contrast demonstrated a well-defined hypervascular lesion involving uncinate process of pancreas measuring 15 mm in diameter with enhancement during the arterial phases of contrast bolus (Fig. 1), but without liver metastasis or intra-abdominal lymph nodes. Magnetic resonance imaging (MRI) of brain was also performed showing an enlarged pituitary gland without nodular lesions. Other hormonal studies including serum cortisol level, parathormone level, adrenocorticotropic hormone (ACTH) level, and thyroid function were normal. Regarding the normal hormonal assessment, multiple endocrine neoplasia was eliminated.

Open surgical exploration was made. Complete mobilization and careful bimanual palpation of the pancreas allowed identifying a firm and well-circumscribed nodule at the uncinate process. Nodule enucleation was performed and pathological examination revealed an encapsulated pancreatic mass measuring 15 × 15 mm. Mitotic index was about 4 per 10 high-power fields (HPF) and proliferation index ki-67 was estimated at 10 %. Immunohistochemically, tumor cells showed a positive and diffuse staining for synaptophysin. Complementary staining for insulin was not preformed because not available in our institution. Therefore, an intermediate grade neuroendocrine tumor of pancreas was identified. Regarding to functional status and biological behavior, diagnosis of pancreatic insulinoma was confirmed. Immediately after surgical treatment, the glucose level increased to the normal range. The patient was discharged without any hypoglycemic symptoms after 4 days. The patient remains asymptomatic within 6 months follow up.

## Case presentation 2

**Fig. 2:**
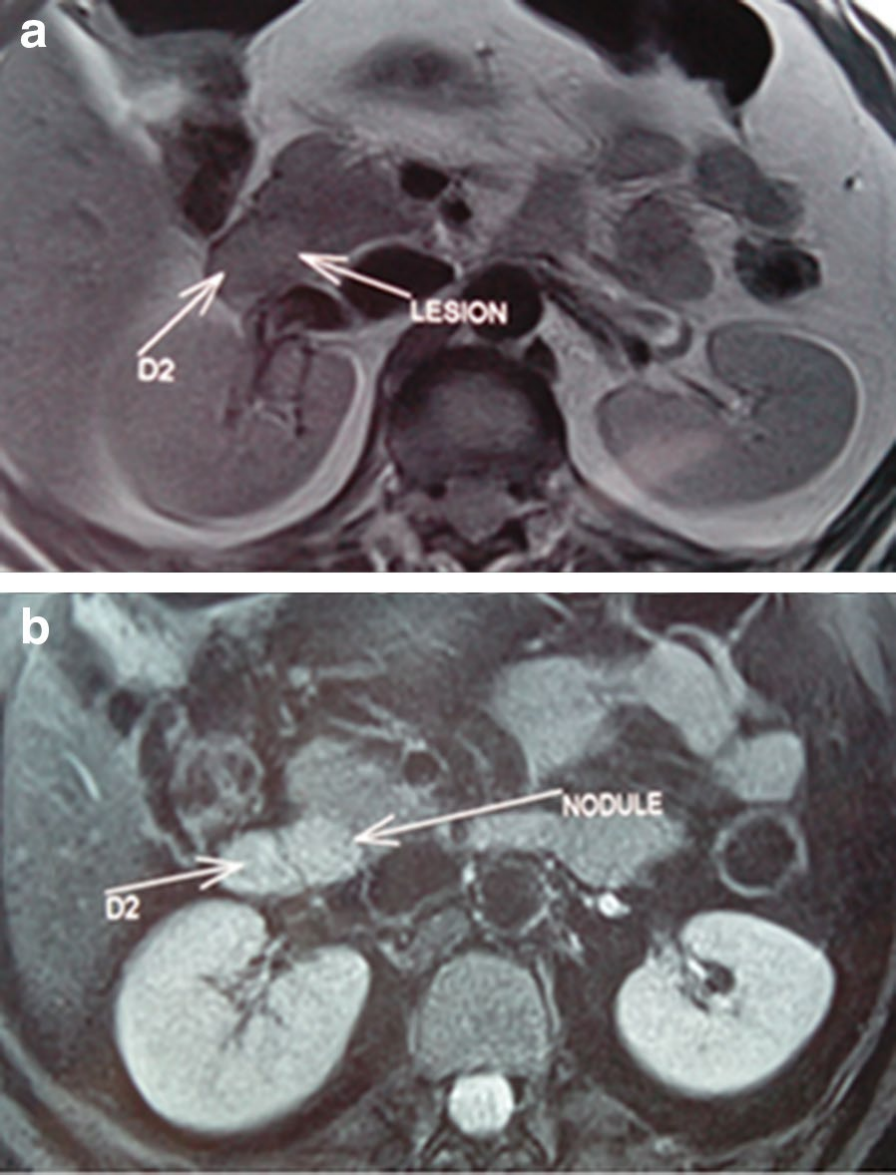
Abdominal magnetic resonance imaging findings: pancreatic nodule with low signal intensity on T1-weighted images (*lesion arrow*) (**a**) and high signal intensity on T2-weighted images (*nodule arrow*) (**b**)

A 47-year-old Arab female suffered from hypoglycemic attacks characterized by dizziness, fatigue, tremulousness, sweating, and hunger, associated with weight gain for 2 years. Most episodes occurred in the evening with a worsening of symptoms during prolonged fasting of Ramadan. She had no family history of endocrine disease. Physical examination showed a well-nourished patient with a BMI of 52.5 kg/m^2^. Blood laboratory tests revealed, a low glucose level at 39 mg/dl (70–11 mg/dl); a high plasma insulin level at 65.2 μIU/ml (2.6–24.9 μIU/ml), and a C-peptide level at 4.99 ng/ml (0.8–4.2 ng/ml). Prolonged supervised fasting test was applied and produced symptomatic hypoglycemia with hyperinsulinemia. Urine for sulfonylurea screen was negative. Abdominal CT scan with contrast demonstrated a hypervascular lesion involving head of pancreas measuring 20 × 17 mm with enhancement during the arterial phases of contrast bolus, but without liver metastasis or intra-abdominal lymph nodes. Abdominal MRI showed a nodule located at the junction head uncinate process of pancreas with intimate relationship to the second portion of duodenum but without bile or pancreatic ducts dilatation (Fig. 2). Thus, endoscopic ultrasonography (EUS) was performed showing a rounded hypoechoic mass of pancreatic head. MRI of hypophysis and cervical ultrasound were normal. Other hormonal studies including serum cortisol level, parathormone level, ACTH level and thyroid function were normal eliminating a multiple endocrine neoplasia.

**Fig. 3:**
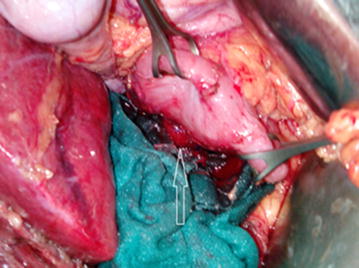
Operative view showing a nodule in the pancreatic head with intimate relationship to the second portion of duodenum (*arrow*)

**Fig. 4:**
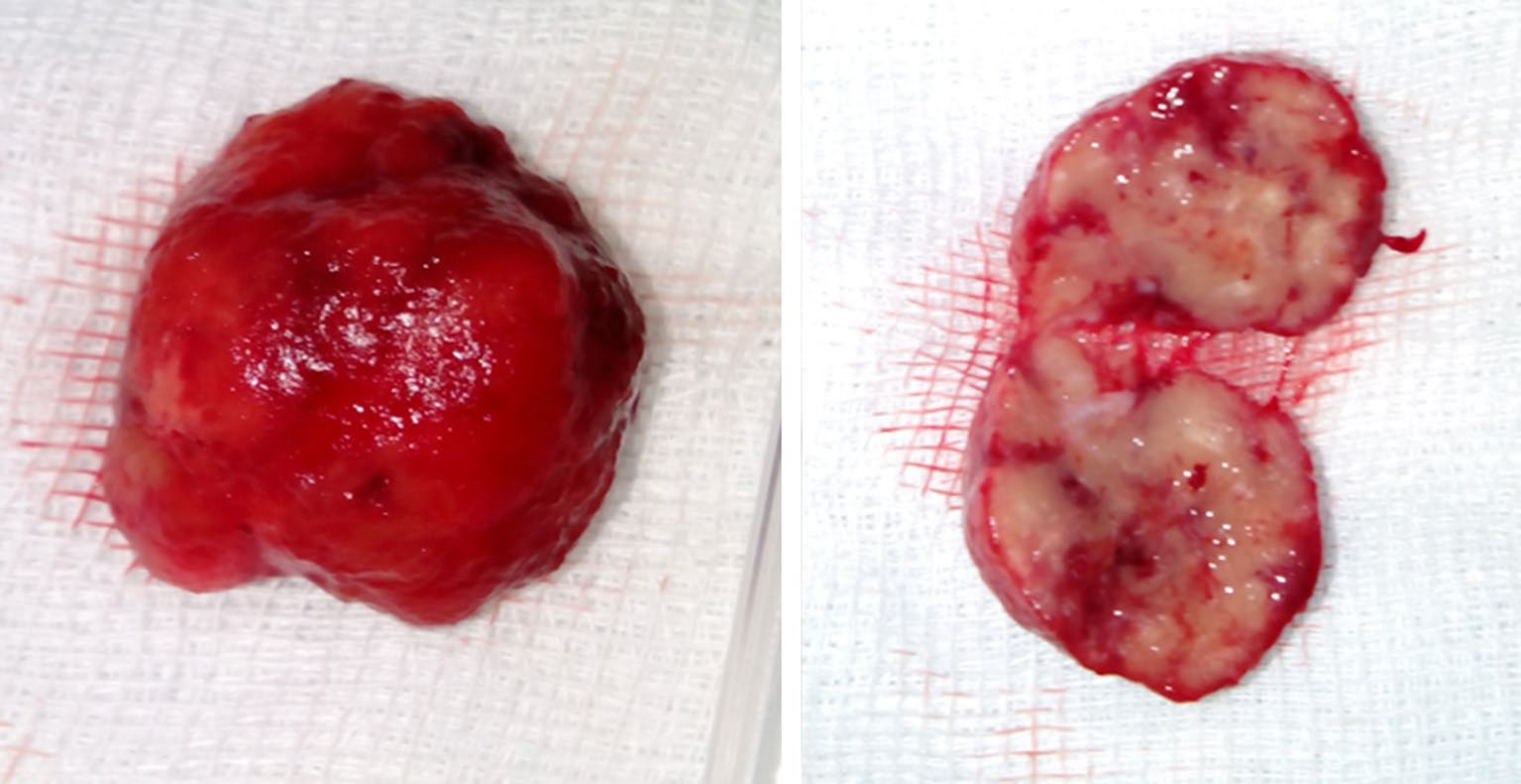
Resected specimen

**Fig. 5:**
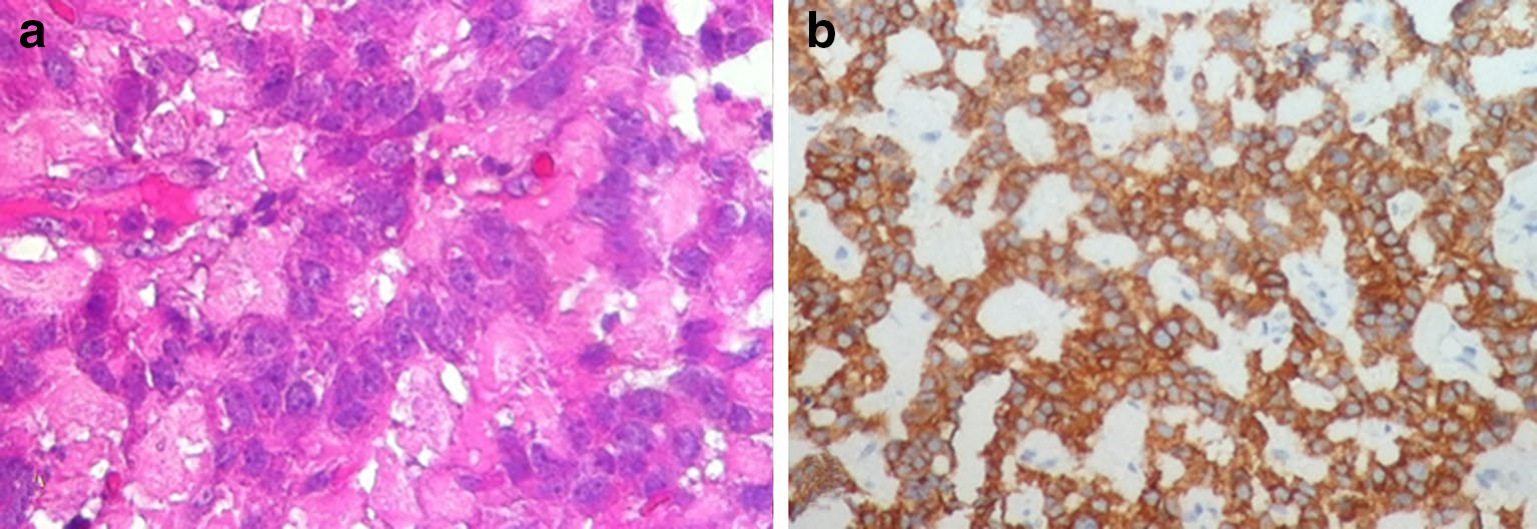
Microscopic findings. **a** Tumor proliferation with endocrine differentiation (hematoxylin-eosin staining, original magnification ×40). **b** Strong and diffuse expression of synaptophysin (immunohistochemistry, original magnification ×100)

Open surgical exploration was made. Complete mobilization and careful bimanual palpation of the pancreas discovered a firm and unifocal nodule in the pancreatic head without relationship to the bile duct. Nodule enucleation was achieved (Fig. 3) and pathological evaluation has revealed an encapsulated pancreatic mass measuring 20 × 15 mm (Fig. 4). Mitotic index was about 2 per 10 HPF and proliferation index ki-67 was estimated at 10 %. Immunohistochemically, tumor cells showed a positive and diffuse staining for synaptophysin (Fig. 5). Complementary staining for insulin was not preformed because not available in our institution. Finally, an intermediate grade neuroendocrine tumor of pancreas was identified. Regarding to functional status and biological behavior, diagnosis of pancreatic insulinoma was confirmed. Immediately after surgical treatment, the glucose level increased to the normal range. The patient was discharged without any hypoglycemic symptoms after 5 days. The patient is currently in 1 year follow-up with a good evolution.

## Discussion

Sporadic distribution, small size and high benignity rate are known insulinoma features, however etiopathogenesis remains still unclear. This rare tumor may have variable and nonspecific presentations all referable to the hypoglycemic state. Hypoglycemic symptoms can be divided into neuroglycopenic signs, most common, including confusion, behavioral changes, visual disturbances, weakness, dizziness, seizures and loss of consciousness, and neurogenic signs, such as anxiety, sweating, palpitations, tremors and feeling of warmth [4, 5].These symptoms become typically evident after fasting and are often precipitated by physical exercises. However, the median duration of symptoms before diagnosis remains variable and can reach 12–18 months on average or even years in rare cases [2]. In our report, insulinoma diagnosis was delayed for several years. Simply because symptoms of hypoglycemia have been misinterpreted and misattributed to cardiac and neurological disorders before the insulinoma was recognized. In addition, our two patients tried to avoid hypoglycemic signs by eating frequently with resultant weight gain.

Insulinoma is still suggested by the Whipple’s triad including: symptoms of hypoglycemia induced by fasting or exercise, plasma glucose level below than 45 mg/dl and relief of symptoms following the administration of glucose. The supervised 72-h fasting test remains to be the gold standard for biochemical diagnosis with measurement of plasma glucose, insulin, C-peptide, and proinsulin during the onset of hypoglycemic symptoms. We used this test in our two cases to diagnose hyperinsulinism.

Various preoperative procedures can be used to localize the tumor in order to plan therapeutic strategy. The choice of procedure depends upon which tests are available and local radiologic skills. In our context, multiple noninvasive and invasive options are used including trans-abdominal ultrasonography, CT scan, MRI and EUS. The reported sensitivity of conventional CT and MRI for detection of pancreatic insulinoma ranges respectively from 33 to 64 and 40 to 90 %. However, the advent of helical CT scan has enabled detection of about 94 % of insulinomas [1, 4]. Consequently, it is currently accepted that CT scan is the first-line and MRI is the second-line investigation. These modalities can identify the exact size and location of an insulinoma, describe its anatomic relationship to surrounding structures and detect the presence of metastatic lesions suggestive of malignancy [6].

Some authors consider EUS as the best exam for preoperative localization of insulinoma, with a sensitivity of up to 94 %. It can detect even small tumors of 5 mm, and reveal important relation to the bile duct and adjacent blood vessels. Also, EUS allows performing fine-needle aspiration cytology of suspicious lesions and preoperative marking of tumors to facilitate surgical excision particularly with laparoscopic approach. However, EUS findings depend largely on the examiner’s experience [1, 2]. In our institution, we generally use this technique when the tumor is not detected with the previously mentioned imaging modalities.

Bimanual palpation combined with intraoperative ultrasonography (IOUS) is the most effective method to detect more than 95 % of tumors, but requires complete mobilization of the pancreas [7, 8]. Because of this operative success particularly in the hands of experienced surgeons, some authors suggest that preoperative localization studies are not necessary [9, 10]. However, with recent advances in imaging techniques, preoperative topographic assessment is considered useful in avoiding blind resection and planning for rapid, accurate and safe surgery. Additionally, there is absolutely no question that positive localization is required prior to re-operative insulinoma [4].

Most insulinomas can be cured with surgery. Surgical procedure choice depends on the size and location of the mass. Tumor enucleation is the procedure of choice especially in case of small and solitary nodule that is not encroaching on the pancreatic or bile ducts [11]. The lesions are typically reddish-brown, firm, and encapsulated with a clear plane of dissection between the tumor and surrounding soft pancreatic parenchyma [12]. In addition, recent guidelines suggest that enucleation is enough in front of a well-circumscribed lesion, clearly localized before surgery, near or at the pancreatic surface, and easily defined intra-operatively [4]. Moreover, pancreatic resection is indicated for lesions invading or in close proximity to the pancreatic duct or major vessels, or suspicious for malignancy with a hard, infiltrating tumor and puckering of the surrounding soft tissue, pancreatic duct dilatation or lymph node involvement [4, 13]. Resection options include distal pancreatectomy (with or without splenectomy), Whipple procedure (pancreaticoduodenectomy), or median pancreatectomy, depending on the site of insulinoma.

If the tumor is not identified despite a careful surgical exploration with bimanual palpation and IOUS, termination of the surgical procedure without blind resection is recommended. In such cases, the patient should be evaluated and re-operated at a referral center. Blind distal pancreatectomy is currently not appropriate and must be avoided [10, 14]. Consequently, more extensive localization procedures must be applied before reoperation, often including the intra-arterial calcium stimulation test with hepatic venous sampling (IACS-test). This test helps to regionalize the lesion preoperatively with a high detection rate ranging from 94 to 100 %. [15]. Therefore, the use of IACS-test allows for a more accurate surgical approach and can minimize the likelihood of re-operation. It may be appropriate when an insulinoma is strongly suspected but all previously described tests are negative. However, high cost and exclusive availability in some specialized centers lead to reserve this modality for patients with persistent or recurrent hyperinsulinism after initial surgery [16].

Furthermore, laparoscopic approach is currently feasible and becomes increasingly reported with good results in selected patients [17, 18]. Tumor location should be confirmed intra-operatively by laparoscopic ultrasonography [19, 20]. Both of our patients underwent tumor enucleation using open surgical approach. The lesions were easily identified thanks to only bimanual palpation of the pancreatic parenchyma.

Histologically, insulinomas are epithelial neoplasms associated with strong and diffuse immunohistochemical expression of neuroendocrine markers such as synaptophysin and chromogranin. Mitotic rate (number of mitoses per 10 HPF) and proliferation index (Ki-67 labeling index) are particularly helpful to separate well-differentiated from poorly differentiated tumors [21]. Conversely, Malignant insulinomas are difficult to distinguish histologically and often the diagnosis of malignancy is only made when metastases occur [10].

Medical management of insulinoma, used to treat and prevent hypoglycemia, is generally restricted to unresectable metastatic tumors, unsuccessful operation with persistent symptoms, inoperable patients, and patients awaiting or refusing surgery [1, 4]. Moreover, other recent techniques for the management of insulinoma have been reported, including injection of octreotide, EUS guided alcohol ablation, radiofrequency ablation, or embolization of an insulinoma [1].

## Conclusion

Insulinoma is a rare neuroendocrine tumor, usually benign, but can be life-threatening in causing hypoglycemic accidents. Biochemical diagnosis is easy, but preoperative localization may prove difficult. However, most insulinomas can be identified intraoperatively by experienced surgeons. Surgical resection remains the treatment of choice with an extremely high success rate. Laparoscopic approach is increasingly performed and blind pancreatic resection is not recommended. Finally, medical options are reserved for unresectable or metastatic tumors.

## Consent

Written informed consent was obtained from both patients for publication of this Case Report and any accompanying images. A copy of the written consent is available for review by the Editor-in-Chief of this journal.

## Abbreviations

MEN-I: multiple endocrine neoplasia syndrome type I
BMI: Body Mass Index
ACTH: adrenocorticotropic hormone
CT: computed tomography
MRI: magnetic resonance imaging
EUS: endoscopic ultrasonography
HPF: high-power fields
IOUS: intraoperative ultrasonography
IACS-test: intra-arterial calcium stimulation test with hepatic venous sampling
